# Distributed Beamforming and Power Allocation for Heterogeneous Networks with MISO Interference Channel

**DOI:** 10.3390/s21082606

**Published:** 2021-04-08

**Authors:** Kisong Lee

**Affiliations:** Department of Information and Communication Engineering, Dongguk University, Seoul 04620, Korea; kisonglee@dongguk.edu; Tel.: +82-2-2260-3233

**Keywords:** heterogeneous networks, MISO interference channel, beamforming, power allocation, distributed algorithm

## Abstract

To address the limitations of centralized resource allocation, i.e., high computational complexity and signaling overhead, a distributed beamforming and power allocation strategy is proposed for heterogeneous networks with multiple-input-single-output (MISO) interference channels. In the proposed scheme, each secondary user transceiver pair (SU TP) determines the beamforming vector and transmits power to maximize its own spectral efficiency (SE) while keeping the interference to the primary user below a predetermined threshold, and such resource management for each SU TP is updated iteratively without any information sharing until the strategies for all SU TPs converge. The simulation confirms that the proposed scheme can achieve a performance comparable to that of a centralized approach with a much lower computation time, e.g., less than 5% degradation in SE while improving computation time by more than 10 times.

## 1. Introduction

With the explosive growth of mobile data traffic and wireless devices, heterogeneous networks emerged as a promising mechanism to provide high data rates and extend communication coverage [1,2,3]. In contrast to those of homogeneous networks, secondary users (SUs) opportunistically share the same spectrum resource as primary users (PUs) in heterogeneous networks, which improves the spectral efficiency (SE), but at the same time, this causes serious cross-tier interference.

Given that the system performance can be improved effectively with dynamic resource allocation [4], a number of studies have been undertaken regarding strategies to efficiently share the spectrum between different networks with suppression of co-channel interference [5,6,7,8,9]. In particular, interference management techniques were investigated to improve the SE of heterogeneous networks in [5,6]. Resource allocation was proposed to jointly maximize the energy efficiency and SE in multicell heterogeneous networks in [7], and joint power control and resource allocation were suggested to maximize the total throughput of cooperative device-to-device (D2D) heterogeneous networks in [8]. Moreover, the authors of [9] devised robust resource management for heterogeneous networks under channel uncertainty.

To further improve upon the performance of heterogeneous networks while mitigating co-channel interference, some attempts have implemented multiantenna techniques, including multiple-input-multiple-output (MIMO) precoding [10] and coordinated scheduling and a beamforming scheme [11]. The analytical expressions of capacity bounds were derived for dense massive MIMO in a line-of-sight propagation environment [12], and the array antennas for MIMO applications were discussed [13,14,15,16,17,18]. Furthermore, the optimal transmit beamforming, power allocation, and bandwidth partitioning were jointly designed to maximize the sum achievable rate of the small cells while protecting the performance of macrocell in [19]. In multiuser and multichannel underlay multiple-input-single-output (MISO) heterogeneous networks, joint beamforming and resource allocation were studied to find the maximum possible number of SUs [20] and to maximize the sum rate of SUs [21] while satisfying the interference requirements for the PUs. In [22,23,24], a distributed beamforming or a power allocation was proposed to improve the performance of cooperative relay networks.

Some previous studies have considered joint optimization for beamforming and resource allocation for heterogeneous networks with multiantenna configurations [11,19,20,21], but they have solved nonconvex optimization problems using a centralized approach, which requires a large signaling overhead to acquire perfect channel state information (CSI) and a high computational complexity. Although a distributed approach was discussed in [22,23,24], it cannot be applied directly to heterogeneous networks. Therefore, it is necessary to devise a distributed approach that can be operated with practical heterogeneous networks.

In this paper, heterogeneous networks are considered with MISO interference channels, in which multiple SU transceiver pairs (TPs) share the same spectrum with the PUs. In such networks, an optimization problem is formulated to find optimal beamforming vectors and transmit powers for the SU TPs to maximize their sum SE with a guarantee of the requirements of allowable interference to the PUs. Given that a centralized approach needs a large signaling overhead and a high computational complexity to determine the suboptimal solutions from the formulated nonconvex problem, a distributed beamforming and power allocation strategy that does not require any information sharing is proposed, where each SU TP determines beamforming vector with the maximum ratio transmission (MRT) and finds transmit power iteratively using dual methods. Simulations in various environments confirm that the proposed scheme accomplishes a performance comparable to the centralized power allocation with MRT in terms of sum SE and violation probability with a remarkable reduction in the computation time.

The remainder of this paper is organized as follows. In Section 2, a system model is presented, together with a formulation of the problem. In Section 3, the distributed beamforming and power allocation strategy is proposed. In Section 4, the performance of the proposed scheme is evaluated under various environments, and finally, the conclusions are presented in Section 5.

## 2. System Model and Problem Statement

**Figure 1:**
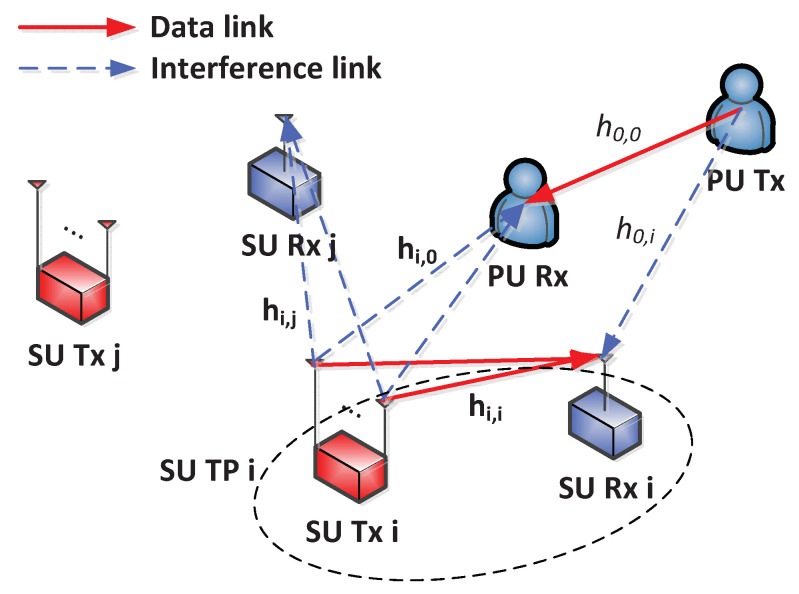
System model of heterogeneous networks with multiple-input-single-output (MISO) interference channels.

Figure 1 shows the system model of heterogeneous networks with MISO interference channels, where there are *N* SU TPs, each of which consists of a transmitter (Tx) equipped with *M* antennas and a receiver (Rx) equipped with a single antenna. The sets of SU TPs and antennas are denoted as N and M, respectively, such that |N|=N and |M|=M. SU TPs share the same spectrum as long as the amount of interference in PUs equipped with a single antenna is less than a predefined threshold. The channel between the Tx of the SU TP *i* and the Rx of the SU TP *j* for antenna *m* is denoted by $h_{i,j}^{\left[m\right]}$, and index 0 is used to indicate the PUs such that $h_{0,i}$ is the channel between the Tx of the PU and the Rx of the SU TP *i*, while $h_{i,0}^{\left[m\right]}$ is the channel between the Tx of the SU TP *i* and the Rx of the PU for antenna *m*. It is assumed that $h_{i,0}^{\left[m\right]}$ for m∈M is available at SU TP *i* to guarantee the requirement for the interference caused to the Rx of the PU [25].

The received signal at the Rx of the SU TP *i* is given by

(1) $$\begin{array}{c} y_{i}=\sqrt{p_{i}}h_{i,i}^{H}w_{i}x_{i}+\sum_{j\in N\backslash \{i\}}\sqrt{p_{j}}h_{j,i}^{H}w_{j}x_{j}+\sqrt{p_{0}}h_{0,i}x_{0}+z_{i}, \end{array}$$

where ${\left(\cdot \right)}^{H}$ indicates Hermitian transpose, $x_{i}$ and $x_{0}$ are the normalized data symbols transmitted by the Tx of the SU TP *i* with transmit power $p_{i}$ and the Tx of the PU with transmit power $p_{0}$, respectively, and $z_{i}∼\mathrm{CN}\left(0,\sigma^{2}\right)$ denotes additive white Gaussian noise. Moreover, $w_{i}$ is a beamforming vector with unit norm, i.e., $w_{i}=\left\{w_{i}^{\left[1\right]},w_{i}^{\left[2\right]},\cdots ,w_{i}^{\left[M\right]}\right\}\in C^{M\times 1}$ and $∥w_{i}{∥}^{2}=1$, and $h_{i,j}=\left\{h_{i,j}^{\left[1\right]},h_{i,j}^{\left[2\right]},\cdots ,h_{i,j}^{\left[M\right]}\right\}\in C^{M\times 1},\forall i,j\in N$.

Then, the achievable SE at SU TP *i* is represented by

(2) $$\begin{array}{cc} r_{i} & =\log_{2}\left(1+\frac{p_{i}{\left|h_{i,i}^{H}w_{i}\right|}^{2}}{\sigma^{2}+p_{0}|h_{0,i}{|}^{2}+\sum_{j\in N\backslash \{i\}}p_{j}{\left|h_{j,i}^{H}w_{j}\right|}^{2}}\right). \end{array}$$

On the other hand, the interference caused from the Tx of the SU TP *i* to the Rx of the PU is expressed as

(3) $$\begin{array}{cc} I_{i} & =p_{i}{\left|h_{i,0}^{H}w_{i}\right|}^{2}. \end{array}$$

The optimization problem can be formulated to find the optimal beamforming vectors and transmit powers of the SU TPs to maximize their sum SE while maintaining the interference level that each SU TP causes to the Rx of the PU at less than the allowable interference level, $I_{\mathrm{th}}$, as follows:

(4) $$\begin{array}{cc} \max_{W,\;0⪯p} & \;\;\;\;\sum_{i\in N}r_{i}\\ s.t. & \;\;\;\;C1:\;\;I_{i}\leq I_{\mathrm{th}},\;\;\;\;i\in N\\ \; & \;\;\;\;C2:\;\;p_{i}\leq P_{\max},\;\;\;\;i\in N\\ \; & \;\;\;\;C3:\;\;∥w_{i}{∥}^{2}=1,\;\;\;\;i\in N, \end{array}$$

where $p=\{p_{1},p_{2},\cdots ,p_{N}\}$, $W=\{w_{1},w_{2},\cdots ,w_{N}\}$, and $P_{\max}$ is the maximum transmit power for each SU TP. However, Problem (4) is nonconvex because of co-channel interference; therefore, it is difficult to find the optimal resource allocation strategy analytically. Although optimal solutions can be found through an exhaustive search, in which W and p are quantized with equispaced values and all possible combinations have been evaluated, it cannot be used in practical systems due to the high computational complexity and signaling overhead for perfect CSI.

## 3. Distributed Beamforming and Power Allocation

To overcome impractical limitations in taking a centralized approach, a distributed beamforming and power allocation strategy is proposed. Given that the optimal beamforming strategy to maximize the SE of each SU TP is the MRT if interference channels are unknown to the SU TP *i*, the beamforming vector for SU TP *i* can be determined as $w_{i}=\frac{h_{i,i}}{∥h_{i,i}∥}$ [26].

Then, (2) can be transformed into

(5) $$\begin{array}{cc} r_{i} & =\log_{2}\left(1+\frac{p_{i}{∥h_{i,i}∥}^{2}}{\sigma^{2}+p_{0}|h_{0,i}{|}^{2}+\sum_{j\in N\backslash \{i\}}p_{j}{\left|g_{j,i}\right|}^{2}}\right), \end{array}$$

where $|g_{j,i}{|}^{2}=\frac{|h_{j,i}^{H}h_{j,j}{|}^{2}}{∥h_{j,j}{∥}^{2}}$. Moreover, (3) can be transformed into

(6) $$\begin{array}{cc} I_{i} & =p_{i}\frac{|h_{i,0}^{H}h_{i,i}{|}^{2}}{∥h_{i,i}{∥}^{2}}\\ \; & =p_{i}{\left|g_{i,0}\right|}^{2}. \end{array}$$

For the determined beamforming vector, an optimization problem is formulated to find the optimal transmit power of the SU TP *i* to maximize its own SE, as follows:

(7) $$\begin{array}{cc} \max_{0\leq p_{i}} & \;\;\;\;r_{i}\\ s.t. & \;\;\;\;C1:\;\;p_{i}{\left|g_{i,0}\right|}^{2}\leq I_{\mathrm{th}}\\ \; & \;\;\;\;C2:\;\;p_{i}\leq P_{\max}. \end{array}$$

Dual methods are applied for distributed power allocation operated in an iterative manner [27]. First, the Lagrangian function of (7) is defined as follows:

(8) $$\begin{array}{cc} L(p_{i},\mu_{i},\lambda_{i}) & =r_{i}+\mu_{i}\left(I_{\mathrm{th}}-p_{i}{\left|g_{i,0}\right|}^{2}\right)+\lambda_{i}\left(P_{\max}-p_{i}\right), \end{array}$$

where $\mu_{i}\geq 0$ and $\lambda_{i}\geq 0$ indicate the Lagrange multipliers for the first and second constraints of (7), respectively, with $\vec{\mu }=\left\{\mu_{1},\mu_{2},\cdots ,\mu_{N}\right\}$ and $\vec{\lambda }=\left\{\lambda_{1},\lambda_{2},\cdots ,\lambda_{N}\right\}$.

Then, its dual objective can be defined as

(9) $$\begin{array}{c} f\left(\mu_{i},\lambda_{i}\right)=\max_{0\leq p_{i}}\;\;L\left(p_{i},\mu_{i},\lambda_{i}\right), \end{array}$$

and the dual problem can be formulated as

(10) $$\begin{array}{c} \min_{0\leq \mu_{i},\;0\leq \lambda_{i}}\;\;f\left(\mu_{i},\lambda_{i}\right). \end{array}$$

On the basis of (9) and (10), $p_{i}$ can be updated to maximize $L(p_{i},\mu_{i},\lambda_{i})$, while $\mu_{i}$ and $\lambda_{i}$ are updated to minimize $f(\mu_{i},\lambda_{i})$ in each SU TP, iteratively.

The Karush–Kuhn–Tucker (KKT) conditions with complementary slackness are also given by

(11) $$\begin{array}{cc} \; & \frac{\partial L}{\partial p_{i}}=\frac{∥h_{i,i}{∥}^{2}}{\ln2\left(p_{i}∥h_{i,i}{∥}^{2}+\sigma^{2}+p_{0}|h_{0,i}{|}^{2}+\sum_{j\in N\backslash \{i\}}p_{j}{\left|g_{j,i}\right|}^{2}\right)}-\mu_{i}{\left|g_{i,0}\right|}^{2}-\lambda_{i}=0 \end{array}$$

(12) $$\begin{array}{cc} \; & \mu_{i}\left(I_{\mathrm{th}}-p_{i}{\left|g_{i,0}\right|}^{2}\right)=0 \end{array}$$

(13) $$\begin{array}{cc} \; & \lambda_{i}\left(P_{\max}-p_{i}\right)=0 \end{array}$$

(14) $$\begin{array}{cc} \; & p_{i}\geq 0,\;\;\mu_{i}\geq 0,\;\;\lambda_{i}\geq 0. \end{array}$$

From (11)–(14), the transmit power that satisfies the KKT conditions can be obtained as

(15) $$\begin{array}{c} p_{i}^{*}={\left[\frac{1}{\ln2\left(\mu_{i}{\left|g_{i,0}\right|}^{2}+\lambda_{i}\right)}-\frac{\Omega_{i}}{∥h_{i,i}{∥}^{2}}\right]}^{+}, \end{array}$$

where ${\left[x\right]}^{+}=\max\left(x,0\right)$ and $\Omega_{i}=\sigma^{2}+p_{0}|h_{0,i}{|}^{2}+\sum_{j\in N\backslash \{i\}}p_{j}{\left|g_{j,i}\right|}^{2}$. It should be noted that $\Omega_{i}$ in (15) indicates the sum of the noise power and interference power from the PU and the other SU TPs; therefore, it can be calculated by subtracting the received signal power from SU Tx i from the total received signal power without information sharing. In other words, SU TP *i* does not need to know the individual value of the parameters in $\Omega_{i}$ to calculate its transmit power.

The following gradient method can be used to update the Lagrange multipliers:

(16) $$\begin{array}{cc} \mu_{i} & \leftarrow {\left[\mu_{i}-\nu \left(I_{\mathrm{th}}-p_{i}{\left|g_{i,0}\right|}^{2}\right)\right]}^{+}, \end{array}$$

(17) $$\begin{array}{cc} \lambda_{i} & \leftarrow {\left[\lambda_{i}-\kappa \left(P_{\max}-p_{i}\right)\right]}^{+}, \end{array}$$

where ν and κ are step sizes that are small enough to guarantee the convergence.

The procedures for the proposed distributed beamforming and power allocation are summarized in Algorithm 1. Specifically, each SU TP initializes the transmit power and the Lagrange multipliers, and then determines its beamforming vector with MRT. Then, it computes transmit power according to (15) and updates the Lagrange multipliers according to (16) and (17) iteratively until the transmit powers for all SU TPs converge. Given that each SU TP does not require any information sharing with other SU TPs to find $w_{i}$ and $p_{i}$ in the proposed algorithm, it can be operated in a distributed manner.
**Algorithm 1** Distributed beamforming and power allocation1: Initialize $p^{\left(0\right)}$, $\vec{\mu }$, and $\vec{\lambda }$, randomly2: Determine $w_{i}=\frac{h_{i,i}}{∥h_{i,i}∥}$,   ∀i∈N3: j←14: **repeat**5:  $p_{\mathrm{old}}\leftarrow p^{\left(j-1\right)}$6:  **for**
i=1 to *N*7:   Compute $p_{i}^{\left(j\right)}$ according to (15)8:   Update $\mu_{i}$ and $\lambda_{i}$ according to (16) and (17)9:  **end for**10:  $p^{\left(j\right)}=\left\{p_{1}^{\left(j\right)},p_{2}^{\left(j\right)},\cdots ,p_{N}^{\left(j\right)}\right\}$11:  j←j+112: **until**
$\left|p^{\left(j\right)}-p_{\mathrm{old}}\right|<\epsilon$


## 4. Simulation Results and Discussion

To evaluate the performance, the following system parameters were chosen based on the underlay D2D communication as default [25,28]: *N* = 2, *M* = 2, $I_{\mathrm{th}}$ = −60 dBm, $P_{\max}=p_{0}$ = 23 dBm, and $\sigma^{2}=-100$ dBm. The nodes are uniformly distributed over an area of 35 m × 35 m with a maximum distance between the Tx and Rx in the same SU TP of 15 m. For the path-loss model, a path-loss exponent of 3.6 is considered and the attenuation at a reference distance of 1 m is set to −30 dB. Moreover, an independent and identically distributed (i.i.d.) circularly symmetric complex Gaussian (CSCG) random variable with zero mean and unit variance is used to generate multipath fading. The following five schemes are considered to evaluate the performance in terms of the sum SE and the probability of violating the constraint of allowable interference on the PUs using AMD Ryzen 9 5950X 16-Core Processor running at 3.40 GHz with 128 GB of memory.

- Optimal (Opt.) power scheme: the beamforming vector is determined by the MRT, i.e., $w_{i}=\frac{h_{i,i}}{∥h_{i,i}∥}$, and an optimal power allocation is found via an exhaustive search in which the transmit power is quantized into 100 equispaced values, and all possible combinations are examined;
- Proposed scheme: a distributed power allocation described in Algorithm 1 is utilized with $w_{i}=\frac{h_{i,i}}{∥h_{i,i}∥}$;
- Equally reduced (ER) power scheme: the same transmit power, $p_{\mathrm{erp}}$, is utilized for each SU TP with $w_{i}=\frac{h_{i,i}}{∥h_{i,i}∥}$, where the optimal value of $p_{\mathrm{erp}}$ is found via an exhaustive search [29];
- Iterative (Iter.) power scheme: the beamforming vector and transmit power are determined similarly to the proposed algorithm without considering the constraint of the allowable interference on PU, e.g., C1 of (7), which is a baseline scheme;
- Random (Rand.) power scheme: randomly generated transmit power is utilized for each SU TP with $w_{i}=\frac{h_{i,i}}{∥h_{i,i}∥}$.

It should be noted that the comparison with the iterative and random power schemes identifies the superiority of adaptive power control while the comparison with the optimal and equally reduced power schemes reveals the effectiveness of the distributed operation of the proposed scheme.

**Figure 2:**
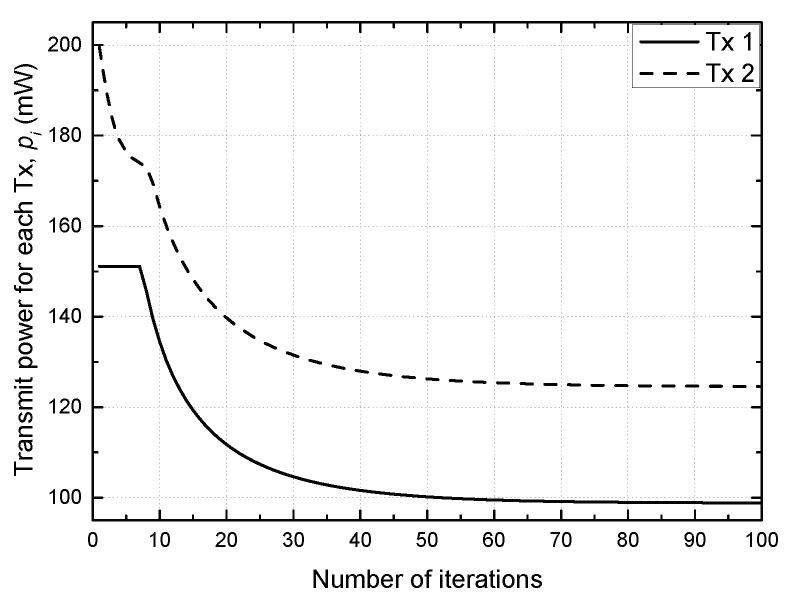
Convergence of the proposed scheme.

Figure 2 shows the convergence of the proposed scheme. Each SU TP determines its transmit power in a direction to maximize its own SE, but it influences the SE of the other SU TP because of interference. In this way, the transmit powers for the SU TPs are updated, affecting each other until they converge. The result confirms that the convergence is made within 60 iterations.

**Figure 3:**
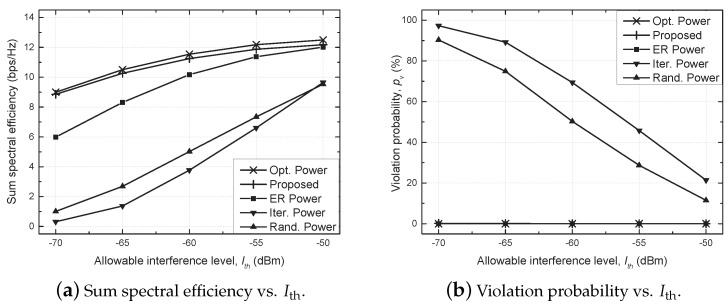
Performance comparison against allowable interference level ($I_{\mathrm{th}}$).

Figure 3 shows the sum SE and violation probability against the allowable interference level ($I_{\mathrm{th}}$) for all considered schemes. It should be noted that the sum SE is set to zero if the constraint for $I_{\mathrm{th}}$ is not satisfied to impose a penalty. The violation probability increases as $I_{\mathrm{th}}$ decreases, because it is more difficult to meet the constraint for $I_{\mathrm{th}}$, which in turn seriously decreases the sum SE for conventional schemes, e.g., the iterative and random power schemes. On the other hand, the proposed scheme satisfies the constraint for $I_{\mathrm{th}}$ well for all range of $I_{\mathrm{th}}$, and it achieves a performance comparable to that of the optimal power scheme and outperforms the equally reduced power scheme. Note that the violation probability is omitted in the following results for brevity, because the effect of the violation is reflected in the sum SE by imposing the penalty.

**Figure 4:**
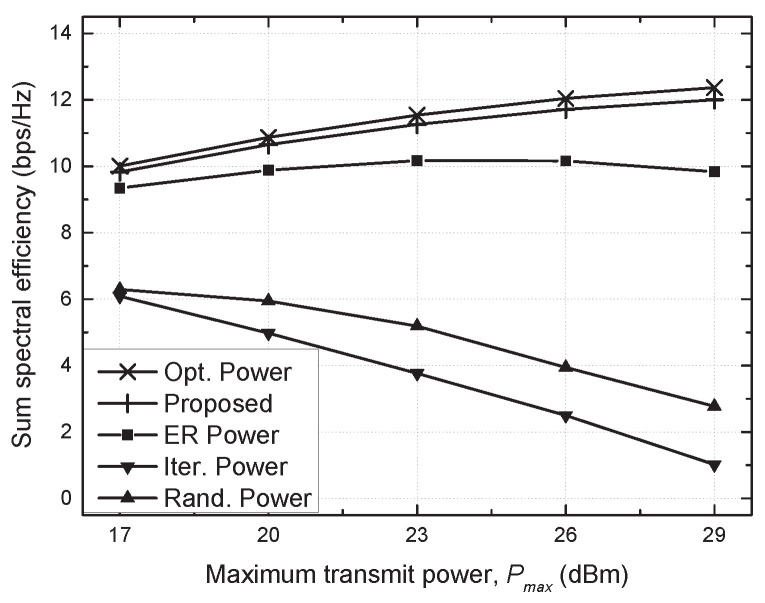
Sum spectral efficiency vs. maximum transmit power ($P_{\max}$).

**Figure 5:**
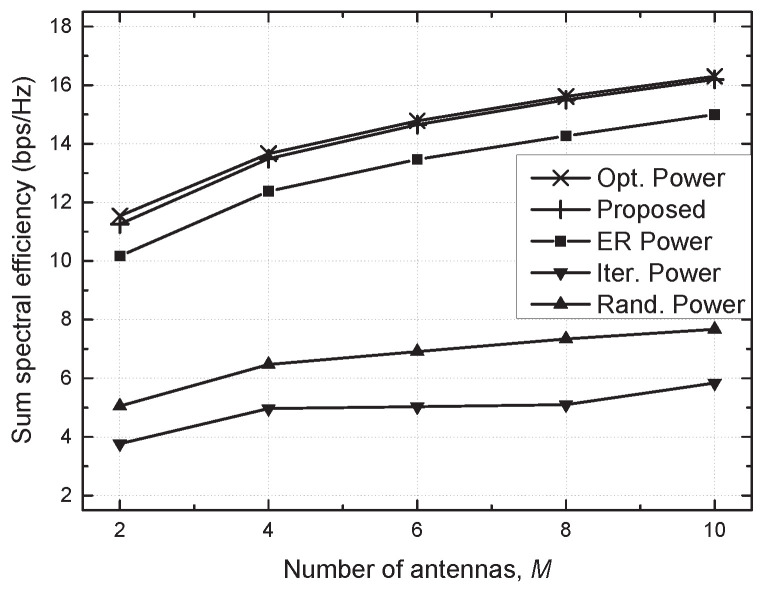
Sum spectral efficiency vs. number of antennas (*M*).

Figure 4 and Figure 5 show the sum SE against the maximum transmit power ($P_{\max}$) and the number of antennas (*M*), respectively. As $P_{\max}$ increases, the sum SE for the conventional schemes decreases due to strong interference. On the other hand, the optimal power and proposed schemes accomplish a higher sum SE with an increasing $P_{\max}$ in virtue of effective resource management. Moreover, the sum SE increases for all schemes as *M* increases due to the antenna diversity. It is also confirmed that the proposed scheme fulfills the performance comparable to the optimal power scheme in these results.

**Figure 6:**
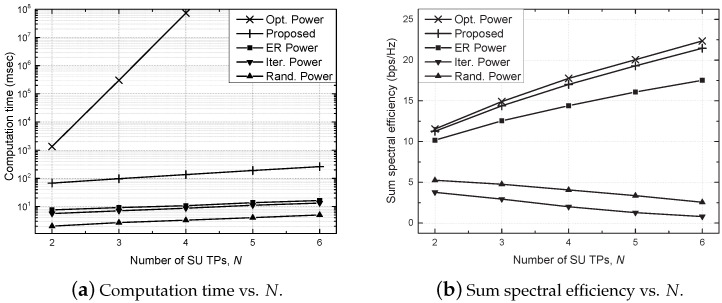
Performance comparison against the number of secondary user transceiver pairs (SU TPs) (*N*).

Figure 6 shows the computation time and sum SE against the number of SU TPs (*N*). As shown in Figure 6a, the computation time of the optimal power scheme increases exponentially with *N*, while the proposed scheme reduces the computation time significantly compared to the optimal power scheme, which confirms the real-time operability of the proposed scheme. It should be noted that the computation time of the random power scheme is very short because these schemes do not perform adaptive power control. Moreover, the equally reduced power scheme can significantly reduce the computation time by reducing the search space from the *N* to 1 dimension although it is based on the centralized approach. Similar to the result in Figure 4, the increased interference degrades the sum SE of the conventional schemes as *N* increases, while the proposed scheme improves the sum SE by allocating the resources properly. Moreover, the sum SE of the proposed scheme approaches that of the optimal power scheme even for a large number of SU TPs. In particular, the proposed scheme shows the degradation of less than 5% in the sum SE while improving computation time by more than 10 times, compared to the optimal power scheme.

## 5. Conclusions

A distributed beamforming and power allocation strategy was investigated for heterogeneous networks with MISO interference channels, where the beamforming vector and the transmit power of each SU TP are determined to maximize its own SE whilst ensuring the requirement of allowable interference on the PUs. In particular, to deal with limitations inherent in the centralized approach, i.e., high computational complexity and high signaling overhead, dual methods were adopted to propose an iterative algorithm operated in a distributed manner without any information sharing. Through intensive simulations, it was confirmed that the proposed scheme outperforms conventional schemes, e.g., more than twice the performance improvement in SE, and achieves a performance comparable to that of the optimal power scheme with a lower computation time, e.g., less than 5% degradation in SE while improving computation time by more than 10 times.

## Data Availability

Not applicable.
